# Changes in fatty acid composition in tissue and serum of obese cats fed a high fat diet

**DOI:** 10.1186/s12917-015-0519-1

**Published:** 2015-08-13

**Authors:** Megumi Fujiwara, Nobuko Mori, Touko Sato, Hiroyuki Tazaki, Shingo Ishikawa, Ichiro Yamamoto, Toshiro Arai

**Affiliations:** Department of Biochemistry and Molecular Biology, Nippon Medical School, 1-1-5 Senndagi, Bunkyou-ku, Tokyo 113-8602 Japan; Department of Veterinary Science, School of Veterinary Medicine, Nippon Veterinary and Life Science University, 1-7-1 Kyonan-cho, Musashino-City, Tokyo 180-8602 Japan

**Keywords:** Cat, GCMS, Total fatty acid, NEFA

## Abstract

**Background:**

Obesity and overweight have been frequently observed in dogs and cats in recent years as in humans. The compositions of fatty acids (FAs) in the accumulated lipids in tissues of obese animals may have important roles in the process and mechanisms related to the onset of metabolic disorders. The purpose of this study was to evaluate the effects of a high fat (HF) diet, which contained a higher proportion of saturated FAs, on FA metabolism and distribution in obese cats.

Cats (*N* = 12) were divided into control diet group (crude fat; 16.0 %) (*n* = 4) or a high fat (HF) diet group (crude fat; 23.9 %) (*n* = 8). The HF diet contained up to 60 % of calories from fat and was rich in stearic acid. Blood samples were collected at 0, 2, 4 and 6 weeks after the feeding. Adipose and liver tissues were collected at the 6^th^ week after feeding. We performed analysis of histological findings and fatty acid composition in serum and tissues.

**Results:**

Body weights of the cats significantly increased in the HF group. The increased activities of hepatic enzymes and the accumulation of lipid droplets were found in hepatocytes in the HF group at the 6^th^ week after feeding.

In this study, the stearic acid (C18:0)-rich HF diet contained less oleic acid (C18:1n-9) and more linoleic acid (C18:2n-6) than the control. However, the composition of oleic acid in the liver was higher, and those of stearic acid and linoleic acid were lower in the HF group at the 6^th^ week after feeding. The higher oleic acid:stearic acid ratio suggests an increase in the conversion from saturated FA to mono-unsaturated FAs, which may reflect the hepatic storage of FAs as a relatively harmless form.

**Conclusion:**

The stearic acid-rich HF diet increased hepatic lipid accumulation accompanied by the increased of hepatic oleic acid, increased serum oleic acid and activation of hepatic enzymes. These findings could be an important sign of early stages of dyslipidemia and hepatic damage. Also, the higher oleic acid:stearic acid ratio might be related to the increased activity of SCD-1, which suggests that the stearic acid-rich HF diet evoked hepatic lipogenesis in the feline liver.

## Background

Obesity and overweight have been frequently observed in dogs and cats in recent years as in humans [1]. Obesity is defined as ectopic lipid accumulation and is a risk factor for metabolic disorders like diabetes, hyperlipidemia, and hypertension in dogs and cats. The compositions of fatty acids (FA) in the accumulated lipids of tissues of obese animals may have important roles in the process and mechanisms related to the onset of metabolic disorders; however, the compositions of FAs are rarely analyzed in the tissues of obese animals. The balance of saturated fatty acids (SFA) and unsaturated fatty acids (UFA) is important because of differences in the effects on FA metabolism and tissue inflammation. For example, SFAs induce metabolic disorders, whereas UFAs have the opposite effect [2, 3]. However, the increase of SFAs in plasma is associated with low-grade inflammation in overweight adolescents, whereas UFAs inhibit pro-inflammatory cytokine production in cells and tissues [4].

There are many similarities between feline and human FA metabolism [5]. In both, FAs are absorbed from the small intestine and are then transported as chylomicrons into the blood. Conversely, endogenously synthesized triglycerides (TG) and cholesterol comprise very low density lipoproteins (VLDLs), and are delivered to peripheral tissue and resolved into non-esterified fatty acids (NEFA) by lipoprotein lipase when necessary. Excess FAs are re-esterified and stored in the liver or adipose tissue [6].

Dietary FA intake can affect the compositions of FAs in the accumulated lipids of the body. FA imbalance could increase inflammation mediators in the tissue and blood. Consumption of a SFA-rich diet has been shown to result in increased inflammation in adipose tissue by regulating lymphocyte receptor signaling [7]. Also, replacement of a SFA-rich diet with UFA-rich diet could decrease plasma inflammatory mediators in humans [8]. There are some similarities in metabolic changes following obesity and diabetes between cats and humans [9]. Like in humans, changes in dietary FAs may be implicated in the disordered lipid metabolism in cats. As yet, there is little information concerning the effects of daily FA compositions on fat metabolism and tissue inflammation in cats.

The purpose of this study was to evaluate the effects of a high fat (HF) diet on FA metabolism and distribution in cats. In this study, we conducted a controlled-feeding trial in which we investigated the effects of a short term feeding of a stearic acid (C18:0)-rich HF diet, which contained a higher proportion of saturated FAs, on the FA composition in the tissue (liver and adipose tissue) and serum in healthy cats.

## Methods

### Animals and diet

The subjects for the experimental protocol were 12 female, mixed-breed cats, which were clinically healthy and cared for in accordance with the guidelines for the care and use of laboratory animals approved by Nippon Veterinary and Life Science University. The cats were divided into two groups: control (*n* = 4) and HF group (*n* = 8). We divided the cats into uneven in case they might refuse to eat the experimental diet. The mean (± SD) age of the control group was 10.0 ± 0.0 months and that of the HF group was 11.5 ± 6.7 months. The mean (± SD) body weight of the control group was 2.4 ± 0.3 kg and that of the HF group was 2.5 ± 0.3 kg. The control group was fed on a commercial diet and the HF group was fed on the HF diet, which was made to order^1^. The commercial diet consisted of fish meal, skim milk, alfalfa, defatted soybean, wheat bran, beet pulp, corn syrup, calcium carbonate, calcium phosphate, salt, iron sulfate, manganese sulfate, zinc carbonate, copper sulfate, vitamins A, D_3_, E, B_1_, B_2_, B_6_, K_3_, and B_12_, niacin, calcium pantothenate, inositol, and folic acid. FA composition of these diets is listed in Table 1. Dietary fats derived from animal sources were used in this study. The HF diet contained up to 60 % of calories from fat; however, the total caloric intakes were same as the control group. We used metabolizable energy calculated with the following formula,

**Table 1:** Composition of nutrients and FA composition in control and HF diets

		Control	HF diet
Composition	Moisture (%)	5.5	7.0
	Crude protein (%)	33.6	32.7
	Crude fat (%)	16.0	23.9
	Crude fiber (%)	3.5	0.9
	Crude ash (%)	5.8	6.8
	Nitrogen free extract (%)	35.9	29.9
	Kcal/g	4.2	4.7
Composition of FAs	C16:0 (%)	35.2	35.5
	C16:1 (%)	7.0	4.9
	C18:0 (%)	14.4	25.0
	C18:1n-9 (%)	34.8	27.6
	C18:2n-6 (%)	5.7	5.0
	C20:4n-6 (%)	2.9	2.0

$$ \mathrm{Metabolizable}\ \mathrm{energy}\ \left(\mathrm{kcal}\right) = \mathrm{Digestible}\ \mathrm{energy}\ \left(\mathrm{kcal}\right)\ \hbox{--}\ \left(0.77 \times \mathrm{g}\ \mathrm{protein}\right) $$

The animal room was maintained at 24 ± 2 °C and at 55 ± 10 % relative humidity on a 12:12 h light:dark cycle (lights on 8:00 AM to 8:00 PM). The cats were housed in individual cages and fed once a day (morning), with water *ad libitum*. Both groups were fed on their foods *ad libitum* for their daily energy requirement (DER) from 9:00 AM to 8:30 AM of the next day. Then, we recorded their food intake. The cats had no access to food for 18 h prior to tests, but free access to water.

Body weight measurements and blood sample collections were repeated at 0, 2, 4 and 6 weeks after feeding. Adipose and liver tissues were collected at the 6^th^ week after feeding. The above operations were performed at 10:00 AM after fasting for 18 h and blood samples were taken from jugular veins.

### Blood sampling and analysis

Peripheral blood was collected into heparinized tubes and serum separation tubes and centrifuged at 1500 × *g* for 10 min; the obtained plasma and serum were stored at −30 °C until use. The plasma biochemical parameters were analyzed using an autoanalyzer^2^. Serum was used for analysis of total FAs and NEFAs with GC/MS.

### Tissue sampling and analysis

The liver, abdominal and subcutaneous adipose tissues were collected at biopsy through a laparoscope. Under anesthesia with propofol, cats underwent a laparoscopic liver biopsy. The liver parenchyma (approximately 5 mm) was cut along the quadrate, right and left medial lobes. At the same time, visceral adipose tissue was obtained from the abdominal cavity and subcutaneous adipose tissues from the inguinal region. Collected tissues were immediately frozen in liquid nitrogen and stored at −30 °C until use. After being freeze-dried, the tissue samples were homogenized and were methylated for the analysis of total FAs.

### Methylation of total FAs in tissue and serum

Total FAs were methylated and extracted using a commercial kit^3^. Dried serum or tissue was incubated with 0.5 mL of reagent A (toluene 52 %, methanol 48 %) and 0.5 mL of reagent B (methanol 93 %) at 37 °C for 60 min. Then, the mixture was kept at 37 °C for 20 min after adding 0.5 mL of reagent C (methanol 30 %). Afterwards, 1.0 mL of extraction reagent (*n*-hexane 96 %) was added to each sample and mixed vigorously and the upper layer was collected. The upper layer was washed with distilled water and injected into washed silica-gel cartridges. After 3.0 mL of wash solvent (*n*-hexane 96 %) was added, 3.0 mL of an eluting solvent (*n*-hexane 96 %, methyl acetate 2 %) was added to elute the FA methyl esters.

### Methylation of NEFAs in serum

After 100 μL of chloroform and 0.1 mM NaOH were added to serum and mixed to extract the NEFA fraction, the upper layer was collected. The collected layer was mixed with an equivalent amount of 0.1 mM HCL for neutralization. A 0.5-mL aliquot of a mixture of chloroform–methanol–water (1:2.5:1, v/v/v) and heptadecanoic acid (C17:0) as internal standard were added to the sample and incubated at 37 °C for 60 min with shaking. Subsequently each sample was centrifuged at 1500 × *g* at 4 °C for 3 min and 50 μL of the lower layer was transferred to a 1.5-mL centrifuge tube. The sample was concentrated to dryness in an evaporator for 30 min. Then the samples were completely dried in a desiccator and derivatized by adding 100 μL of 3 % hydrochloric-methanol at 30 °C for 2 h. Samples were dried again under nitrogen gas, and then 200 μL of hexane was added.

### FA composition with GC/MS analysis

Analysis of FA composition was performed using a GC/MS system^4^. A DB-5 MS capillary column (30 m × 0.25 mm × 0.25 μm^5^) was used to analyze derivatized samples. Helium was used as the carrier gas at 1.0 mL/min. An injection volume of 1 μL was used and the injector and source temperature was 280 °C. The column oven temperature was programmed from 40 to 320 °C at 6 °C/min and held at 40 and 320 °C for 2 and 1 min, respectively. To detect and eliminate retention time shifts, a standard alkane series mixture (C-10 to C-40) was injected periodically into the GC/MS systems. Retention time correction of peaks based on retention time of the standard alkane series mixture was performed using the AART (Automatic Adjustment of Retention Time) function of the GC/MS solution software (Shimadzu, Milan, Italy). Chromatogram acquisition and compound identification by a mass spectral library search were performed using the Shimadzu GC/MS solution software.

### Histological observation of livers of animals

A small amount of liver was taken from anaesthetized animals by laparotomy and fixed in 10 % buffered formalin and embedded in paraffin. Sections of livers were stained with hematoxylin and eosin, and with oil red O to investigate lipid accumulation in livers.

### Statistics

Statistical analysis of the data was performed with GraphPad Prism software version 5. The data are expressed as means ± SD. Statistical processing was performed for the transition of the total FA and NEFA concentrations and biochemical parameters with two-way ANOVA, followed by the Bonferroni *post hoc* tests. Differences between groups were considered significant at *P* < 0.05.

## Results

**Fig. 1:**
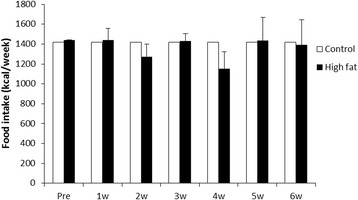
Average weekly food consumption of cats. *Open bars*: Control, *Closed bars*: High fat group

We recorded the food consumption data (Fig. 1). There was no difference in food intake between the two groups.

### Clinical findings

**Fig. 2:**
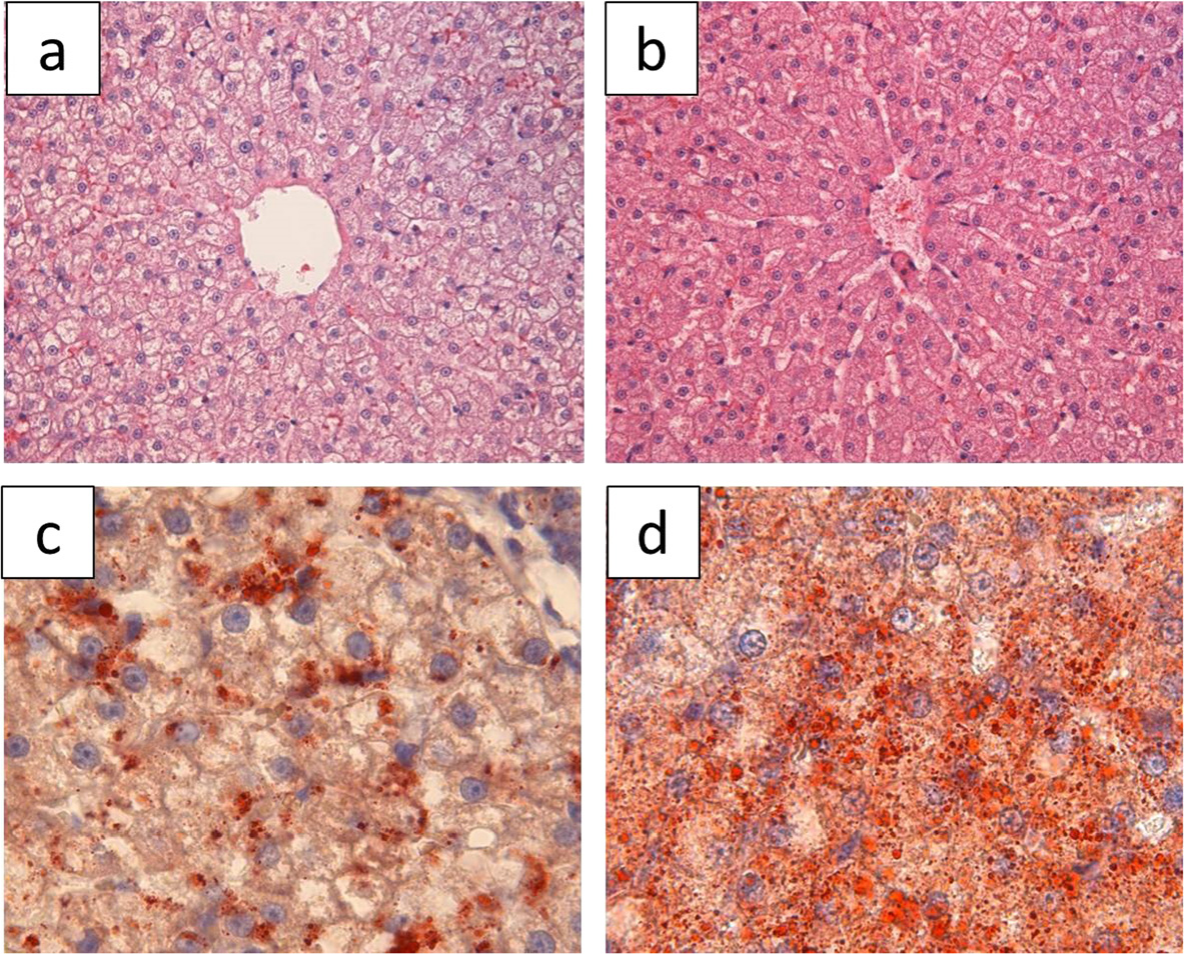
Histology of livers of cats after feeding. Hematoxylin and eosin (HE) staining of livers from the control group (**a**) and HF group (**b**). Oil red O staining of livers (**c** and **d**) revealed the accumulation of lipid droplets in hepatocytes in the HF group. Cells in Fig. 2a appeared to have greater steatosis than cells in Fig. 2b

Body weights of the cats significantly increased in the HF group (from 2.6 ± 0.3 to 3.2 ± 0.5 kg; *P* < 0.01), whereas it did not change in the control group (from 2.5 ± 0.3 to 2.7 ± 0.3 kg). The plasma concentrations of aspartate aminotransferase (AST), alanine aminotransferase (ALT) activities and glucose concentrations increased significantly in the HF group at the 6^th^ week after feeding (*P* < 0.05, Table 2). In the histological observations, the accumulation of lipid droplets was found in hepatocytes in the HF group (Fig. 2).

**Table 2:** Changes in plasma biochemical parameters in cats fed a control or HF diet for 6 weeks

		Pre			2 weeks			4 weeks			6 weeks		
Cont (*n* = 4)	TP (g/dl)	6.5	±	0.4	6.4	±	0.7	6.7	±	6.8^*^	7.0	±	0.5^*^
	AST (IU/l)	26.5	±	1.7	32.5	±	3.7	29.0	±	1.7	30.0	±	1.8
	TG (mg/dl)	44.1	±	17.3	49.5	±	3.5	38.4	±	7.0	50.0	±	10.0
	ALT (IU/l)	52.0	±	11.9	61.5	±	5.7	65.0	±	13.5	57.0	±	11.4
	ALP (IU/l)	96.5	±	29.4	170.0	±	62.5^**A^	114.5	±	33.5^A^	119.0	±	40.3
	LDH (IU/l)	107.0	±	25.3	116.5	±	12.5	109.5	±	80.0	112.0	±	40.7
	T-cho (mg/dl)	106.0	±	8.8	99.5	±	8.0	116.0	±	11.9	104.5	±	8.7
	Glu (mg/dl)	76.5	±	6.5	79.5	±	3.9	79.5	±	26.3	77.0	±	1.3
	BUN (mg/dl)	23.5	±	1.3	22.5	±	1.7	22.5	±	1.5	22.5	±	1.7
	Crea (mg/dl)	0.7	±	0.1	0.9	±	0.2	0.9	±	0.1	0.9	±	0.1
	NEFA (mEq/L)	0.4	±	0.0	0.5	±	0.0	0.3	±	0.1	0.5	±	0.4^*^
HF (*n* = 8)	TP (g/dl)	6.5	±	0.6	6.4	±	0.6	6.5	±	0.5	6.7	±	0.5
	AST (IU/l)	27.5	±	4.5	30.5	±	6.4^*^	31.5	±	5.6^*^	32.0	±	4.5^**^
	TG (mg/dl)	44.1	±	15.5	40.3	±	19.8	40.3	±	7.5	32.4	±	29.7
	ALT (IU/l)	41.0	±	14.2	59.5	±	10.5	61.5	±	12.7^*^	70.5	±	12.4^**^
	ALP (IU/l)	77.0	±	50.6	200.0	±	16.2^**BC^	112.0	±	52.2^B^	109.5	±	55.0^C^
	LDH (IU/l)	136.0	±	46.3	122.0	±	54.2	112.5	±	46.4	126.5	±	68.3
	T-cho (mg/dl)	104.0	±	20.2	93.5	±	21.4^**^	98.0	±	17.5	103.0	±	29.1
	Glu (mg/dl)	73.0	±	5.7	81.0	±	7.8	84.5	±	15.1^**^	76.0	±	10.6
	BUN (mg/dl)	23.5	±	3.8	22.5	±	2.2	23.5	±	2.5	22.5	±	2.3^**^
	Crea (mg/dl)	0.8	±	0.3	0.9	±	0.2	1.0	±	0.2	1.1	±	0.2^**^
	NEFA (mEq/L)	0.4	±	0.1	0.4	±	0.1	0.3	±	0.1	0.6	±	0.2^*^

Significantly different from Pre: ^*^; *P* < 0.05 ^**^; *P* < 0.01. The same capital letters indicate statistically significant differences between the time points (*P* < 0.05)

### FA composition in total FAs in liver

The hepatic FA composition at the 6^th^ week after feeding was different between the groups (Table 3). The composition of oleic acid (C18:1n-9) [% of total FA] was higher (27.9 ± 3.3 % vs. 13.0 ± 0.2 %), and those of stearic acid (C18:0) and linoleic acid (C18:2n-6) [% of total FA] were lower in the HF group (13.2 ± 0.1 % vs. 20.5 ± 3.74 %; 31.5 ± 1.6 % vs. 39.2 ± 1.9 %, respectively).

**Table 3:** FA composition in serum total FAs of cats fed a control or HF diet for 6 weeks

		Pre			2 week			4 week			6 week		
		(%)			(%)			(%)			(%)		
Control (*n* = 4)	C16:0	10.7	±	1.1	9.5	±	0.9	9.8	±	1.1	10.5	±	0.6
	C16:1n-7	6.6	±	1.3	7.3	±	0.3	7.1	±	0.5	7.2	±	0.4
	C18:0	14.8	±	0.9	16.1	±	1.3	15.6	±	1.1	14.5	±	0.3^a^
	C18:1n-9	20.6	±	3.2	20.8	±	3.1	20.0	±	2.4^b^	19.8	±	0.8^c^
	C18:2n-6	38.6	±	2.3	38.5	±	3.1	39.4	±	4.2^d^	40.5	±	1.4^e^
	C20:4n-6	7.7	±	0.3	7.3	±	0.8	8.5	±	2.1	6.7	±	0.7^f^
HF (*n* = 8)	C16:0	10.5	±	0.6	10.1	±	0.8	10.2	±	0.8	10.8	±	1.1
	C16:1n-7	5.9	±	1.4	7.7	±	2.7	6.4	±	2.0	6.4	±	1.7
	C18:0	14.7	±	1.0	14.9	±	1.1	15.5	±	0.7	15.5	±	0.9^*a^
	C18:1n-9	18.8	±	2.0	28.1	±	3.4^**^	25.1	±	1.0^**b^	24.6	±	1.6^**c^
	C18:2n-6	34.4	±	3.8	31.3	±	4.7^*^	32.0	±	2.9^*d^	31.0	±	2.8^**e^
	C20:4n-6	12.2	±	4.6	8.7	±	1.4^**^	11.2	±	1.6	10.5	±	2.0^f^

Significantly different from Pre: ^*^; *P* < 0.05 ^**^; *P* < 0.01. The same lowercase letters indicate statistically significant differences between the two groups (*P* < 0.05)

### FA composition in serum total FAs and NEFAs

The composition of oleic acid (C18:1n-9) [% of total FA] in serum total FAs in the HF group increased from the 2^nd^ week after feeding (*P* < 0.01). The linoleic acid (C18:2n-6) composition [% of total FA] in serum total FAs decreased (*P* < 0.05) and that of arachidonic acid (C20:4n-6) tended to decrease from the 2^nd^ week after feeding (Table 4). In terms of the compositions between the two groups, the component of oleic acid was significantly greater (*P* < 0.05, *P* < 0.01), whereas that of linoleic acid was significantly lower (*P* < 0.05, *P* < 0.01) in HF group at the 4^th^ and 6^th^ week, respectively, after feeding. Arachidonic acid was significantly greater only at the 6^th^ week (*P* < 0.01, Table 4). In terms of the FA composition of serum NEFAs, the linoleic acid and arachidonic acid compositions [% of total NEFA] decreased in the HF group after feeding (Table 5). However, compositions of palmitic acid (C16:0) and oleic acid [% of total FA] increased at the 6^th^ week after feeding.

**Table 4:** Changes in FA composition in total FAs in the liver of cats fed a control or HF diet

	Control (*n* = 2)			HF (*n* = 2)		
C16:0 (%)	11.5	±	1.5	13.8	±	1.0
C16:1n-7 (%)	2.0	±	0.1	1.4	±	0.1
C18:0 (%)	20.5	±	3.7	13.2	±	0.1
C18:1n-9 (%)	13	±	0.2	27.9	±	3.3
C18:2n-6 (%)	39.2	±	1.9	31.5	±	1.6
C20:4n-6 (%)	13.8	±	0.4	12.2	±	0.7

**Table 5:** Changes in FA composition in NEFAs in the serum of cats fed a control or HF diet

		Pre			2 weeks			4 weeks			6 weeks		
		(%)			(%)			(%)			(%)		
Control (*n* = 4)	C16:0	21.0	±	1.0	20.0	±	2.8	20.9	±	1.8	21.5	±	2.7
	C18:0	37.8	±	1.6	32.9	±	5.4	36.4	±	2.3	39.8	±	4.5
	C18:1n-9	5.8	±	0.4	5.7	±	0.6^a^	4.9	±	0.5	4.8	±	0.5^b^
	C18:2n-6	32.9	±	0.9	39.4	±	2.6^**^	35.5	±	1.0^c^	32.7	±	2.7
	C20:4n-6	2.1	±	0.3	1.6	±	0.2	1.4	±	0.3	1.1	±	0.6
HF (*n* = 8)	C16:0	20.0	±	2.5	18.8	±	2.0^AB^	22.8	±	2.8^A^	22.7	±	5.2^*B^
	C18:0	34.3	±	2.2	37.4	±	5.10	43.0	±	3.1^*^	36.5	±	8.7
	C18:1n-9	6.0	±	0.6	8.7	±	1.8^**Ca^	5.8	±	1.1^CD^	8.2	±	1.5^**Db^
	C18:2n-6	37.0	±	2.1	33.8	±	5.0 ^E^	28.3	±	3.7^**Ec^	30.2	±	6.1^**^
	C20:4n-6	2.6	±	1.0	1.7	±	0.5^**^	1.6	±	0.5^**^	1.8	±	0.8^**^

Significantly different from Pre: ^*^; *P* < 0.05 ^**^; *P* < 0.01. The same capital letters indicate statistically significant differences between the time points (*P* < 0.05). The same lowercase letters indicate statistically significant differences between the two groups (*P* < 0.05)

### FA composition in total FAs in adipose tissue

The FA composition in total FAs in adipose tissue at the 6^th^ week after feeding was not different between groups. Additionally, there was no difference in FA composition between abdominal and subcutaneous fat (Table 6).

**Table 6:** Changes in FA composition in total FAs in the adipose tissue of cats fed a control or HF diet for 6 weeks

		Control (*n* = 2)			HF (*n* = 2)		
SAT	C16:0 (%)	27.0	±	0.3	27.2	±	1.5
	C16:1n-7 (%)	0.3	±	0.0	1.3	±	1.5
	C18:0 (%)	12.3	±	0.8	12.1	±	0.1
	C18:1n-9 (%)	59.0	±	0.8	57.9	±	0.6
	C18:2n-6 (%)	1.0	±	0.2	1.0	±	0.4
	C20:4n-6 (%)	0.4	±	0.1	0.3	±	0.1
VAT	C16:0 (%)	26.1	±	0.6	26.1	±	3.5
	C16:1n-7 (%)	0.4	±	0.1	0.4	±	1.5
	C18:0 (%)	11.8	±	2.0	11.8	±	1.0
	C18:1n-9 (%)	60.6	±	2.4	60.6	±	3.5
	C18:2n-6 (%)	0.7	±	0.1	0.7	±	0.4
	C20:4n-6 (%)	0.4	±	0.0	0.4	±	0.1

SAT: subcutaneous adipose tissue, VAT: visceral adipose tissue

## Discussion

The histological findings showed lipid accumulation, which mainly consisted of TGs, in hepatocytes from the HF group. When hepatic FA oxidation is less than its influx, FAs may be accumulated or elongated [10]. These results were consistent with the study, which showed that HF feeding induced the accumulation of hepatic TGs without causing blood TG levels to increase in mice [11]. Though there is a report in which increased caloric intake caused fat storage in the feline liver [12], there are no studies about the effects of a high-fat diet on hepatic steatosis in cats. The plasma biochemical analysis showed significant increases in AST and ALT activity levels in the HF group after feeding in this study. Felines with hepatic lipidosis have been shown to have significantly higher activity levels of ALT and AST in the plasma [13]. Increased ALT and AST activity levels are one of the most common indicators of liver damage. These findings suggested that HF feeding triggered an early stage of hepatic lipidosis and damage in the cats. Also, the increased serum adiponectin concentrations in cats with hepatic lipidosis could be related to hepatic damage, may be the result of hepatic resistance to adiponectin [14]. Adiponectin is most abundantly produced by adipose tissue [15] and increases fat combustion and energy consumption. When the sensitivity to adiponectin is reduced, it may lead to fat accumulation.

In this study, an increased composition of oleic acid and decreased stearic acid was observed after feeding in the HF group. These results are in agreement with reports that SFA and monounsaturated fatty acids (MUFAs) increased in cats with idiopathic fatty liver and humans with nonalcoholic fatty liver disease or diabetes [16–18] The major sources of FAs for hepatic TGs were chylomicron remnants from dietary fat, derivation from adipose tissue, and *de novo* synthesis from the liver [13]. In this study, the stearic acid-rich HF diet contained less oleic acid and more linoleic acid than the control, which did not correlate with the hepatic accumulated FA composition in the HF group. Also, there were different contents of FAs in the liver and adipose tissue of the HF group after feeding. These results did not support the hypothesis that the origin of increased oleic acid accumulation in the liver was derived from the chylomicron remnants from dietary fat or mobilization from adipose tissue. The higher oleic acid:stearic acid ratio could be related to the increased activity of Δ9 desaturase (SCD-1), which catalyzes the conversion of palmitic acid (16:0) and stearic acid (18:0) into palmitoleic (16:1n-7) and oleic acid [19]. SCD-1 activity was reported to increase with increasing liver fat contents [20]. It has been thought that little lipogenesis occurred in the feline liver [17]. However, the increase of oleic acid, one of the major products of lipogenesis, suggested that the stearic acid-rich HF diet evoked hepatic lipogenesis in the cats. SCD-1 has a protective effect against SFAs lipotoxicity by enhancing conversion of dietary SFAs to MUFAs [21]. In this study, the increase in conversion from SFAs to MUFAs may reflect the hepatic safe storage of FAs as a relatively harmless form.

In this study, the FA composition of hepatic and serum FA showed greater percentages of oleic acid and lesser percentages of linoleic acid. In fasting serum, most of the TGs were contained in VLDL and its proportion changed along with increasing adiposity. Actually, it was reported that VLDL contained 30 % of TGs in lean cats, whereas it was increased to 70 % in obese cats [22]. Another study about feline hepatic lipidosis showed that VLDL assembly and secretion were increased with hepatic lipidosis [23]. Also, it was thought that increased oleic acid in NEFAs also stimulate hepatic VLDL production [24]. In the present study, we did not perform an analysis of changes in lipoproteins. However, considering the similarities between the FA composition of the serum and liver, it was suggested that changes in the composition of serum FAs could be reflected by the FAs in the VLDL. To clarify these etiologies, further study is needed to investigate the amount and composition of the VLDL-TGs. Another trend, the changes in the composition of total FAs in serum, was in parallel with those of NEFAs. It was reported that only 4–7 % of plasma NEFAs are directed toward VLDL-TGs under post-absorptive conditions in healthy men and women [25, 26]. However, in overweight/obese man, it is thought that hepatic FA oxidation was decreased, which diverted twice the amount of NEFAs (15 %) toward hepatic VLDL-TG esterification and secretion [27]. Thus, the changes in serum NEFA composition could reflect the VLDL composition in the HF group cats. In this study, the exact mechanisms regarding the change in NEFA composition were not clear. The majority of plasma NEFAs was remobilized from adipose tissue by hormone-sensitive TG lipase activation [28]. However, the different contents of the FAs in plasma NEFAs and adipose tissue did not support the hypothesis that NEFAs were mobilized from adipose tissue in this study. Further analysis may be necessary that include examining the relationship between the origin of NEFAs and VLDL composition.

### Limitation

There are some limitations in this study related to the diets and the sampling of tissues. First, the control diet contained higher amounts of fiber. This might have an effect on the efficiency of fat absorption in the intestine. The contents of C16:1, C18:1n-9, C18:2n-6, and C20:4n-6 in the HF diet were decreased compared with the control diet to create a higher ratio of stearic acid. Second, the tissue samples were obtained from only two cats per dietary treatment group.

## Conclusion

A stearic acid-rich HF diet increased hepatic lipid accumulation accompanied by the increased ratio of hepatic oleic acid, increased serum oleic acid, and activation of hepatic enzymes. These findings could be an important sign of early stages of dyslipidemia and hepatic damage. Also, the higher ratio of oleic acid:stearic acid might be related to the increased activity of SCD-1, which suggests that a stearic acid-rich HF diet evoked hepatic lipogenesis in the feline liver.

## Abbreviations

SFA: Saturated fatty acid
UFA: Unsaturated fatty acid
FA: Fatty acid
HF: High fat
NEFA: Non-esterified fatty acid
TG: Triglyceride
VLDL: Very low density lipoprotein
MUFA: Monounsaturated fatty acid
TP: Total protein
AST: Aspartate aminotransferase
ALT: Alanine aminotransferase
ALP: Alkaline phosphatase
LDH: Lactate dehydrogenase
T-cho: Total cholesterol
Glu: Glucose

## Footnotes

1 Nippon Pet Food, Tokyo, Japan.

2 AU680, Beckman Coulter, Fullerton, CA

3 Nacalai Tesque, Kyoto, Japan

4 Shimadzu QP2010 GC/MS system, Shimadzu, Kyoto, Japan

5 J & W Scientific, Folsom, CA, USA
